# Diabetes and Stroke: Impact of Novel Therapies for the Treatment of Type 2 Diabetes Mellitus

**DOI:** 10.3390/biomedicines12051102

**Published:** 2024-05-16

**Authors:** Inês Henriques Vieira, Tânia Santos Carvalho, Joana Saraiva, Leonor Gomes, Isabel Paiva

**Affiliations:** 1 Department of Endocrinology Diabetes and Metabolism, Hospitais da Universidade de Coimbra—ULS Coimbra, 3004-561 Coimbra, Portugal; 17690@ulscoimbra.min-saude.pt (T.S.C.);; 2 Faculty of Medicine, Universidade de Coimbra, 3004-531 Coimbra, Portugal

**Keywords:** stroke, diabetes type 2, glucagon-like peptide 1 receptor agonists, sodium–glucose cotransporter type 2 inhibitors

## Abstract

Type 2 diabetes mellitus (T2DM) is a significant risk factor for stroke. Nevertheless, the evidence supporting stringent glycemic control to reduce macrovascular complications, particularly stroke, is not as clear as for microvascular complications. Presently, risk reduction strategies are based on controlling multiple risk factors, including hypertension, dyslipidemia, glycemia, smoking, and weight. Since 2008, new pharmacological therapies for treating T2DM have been required to undergo trials to ensure their cardiovascular safety. Remarkably, several novel therapies have exhibited protective effects against the combined endpoint of major cardiovascular events. Evidence from these trials, with stroke as a secondary endpoint, along with real-world data, suggests potential benefits in stroke prevention, particularly with glucagon-like peptide 1 receptor agonists. Conversely, the data on sodium–glucose cotransporter type 2 inhibitors remains more controversial. Dipeptidyl peptidase 4 inhibitors appear neutral in stroke prevention. More recent pharmacological therapies still lack significant data on this particular outcome. This article provides a comprehensive review of the evidence on the most recent T2DM therapies for stroke prevention and their impact on clinical practice.

## 1. Introduction

Stroke is the second most common cause of death and a leading cause of adult disability, affecting approximately 1.1 million inhabitants in the European Union every year [1].

In patients with diabetes mellitus (DM), the risk of stroke increases at least 1.5–3 times, with previous data reporting an increase of more than 4 times in men and 5 times in women. The risk of ischemic stroke escalates significantly with DM duration, with estimates indicating a 3% increased risk for each year of the disease, and a threefold higher risk among those with DM for over 10 years [2]. The prevalence of DM is more common in people with ischemic than hemorrhagic stroke—33 vs. 26%, respectively [3].

Most risk reduction strategies are based on controlling multiple risk factors, such as high blood pressure, dyslipidemia, glycemic control, smoking cessation, and weight loss. Currently, there are few therapies available that specifically target the risk of stroke [3].

In this narrative review, we sought to gather comprehensive data on the effect of the most recent non-insulin pharmacological therapies used in the treatment of diabetes on the risk of stroke and to translate it into recommendations for clinical practice. To this end, we performed a search on the PubMed database combining the medical subject heading (MESH) terms “Diabetes Mellitus, Type 2” and “Stroke”. Articles with full text in English that provided information on the study question were selected based on their title and/or abstract. We prioritized articles published in the last 10 years; however, when particularly relevant or to provide context, older publications were also included. The bibliographies of the initially selected publications were also scanned, with the inclusion of other relevant texts. Additional research was conducted to provide context and to answer questions that emerged during the peer-review process.

## 2. Pathophysiology of Stroke in Diabetes Mellitus Patients

Due to its impact on atherosclerosis development, DM stands out as a major vascular risk factor. Nevertheless, unlike ischemic heart disease, where arteriosclerosis is key, stroke’s pathogenic mechanisms are more complex. Only about 20–25% of all ischemic strokes are derived from atherosclerosis. Other significant mechanisms include cardioembolic origin (30%), small vessel disease (25%), and various rare causes, such as hypercoagulable states and inflammatory and noninflammatory vasculopathy. Furthermore, approximately 20% of strokes are hemorrhagic. DM heightens the risk of both ischemic and hemorrhagic strokes, underscoring the complexity of stroke prevention in this population [4].

In addition to its contribution to atherosclerosis, diabetes directly heightens the risk of cardioembolic events. It has been reported that, in atrial fibrillation (AF) patients, the presence of diabetes alone increases the risk of stroke by 4.0% a year [5].

The underlying pathophysiology of stroke and type 2 DM (T2DM) is also complicated by the association of other risk factors for stroke frequently seen in people with T2DM, such as hypertension and dyslipidemia [3].

At the microvascular level, four main mechanisms seem to predispose to stroke in patients with DM: acidosis, oxidative stress, inflammation, and changes in mitochondrial function. Theoretically, anaerobic conditions in areas of ischemia result in lactate accumulation, which can generate acidemia, which is exacerbated by hyperglycemia. However, it is still controversial whether it is acidemia per se or the combination of acidemia and hyperglycemia that causes harmful results [6].

Oxidative stress ensues when there is an imbalance between reactive oxygen species (ROS) production and the organism’s antioxidant defenses, and is a major contributor to diabetes chronic complications. It may lead to cell death through several mechanisms, including protein denaturation, enzyme inactivation, and deoxyribonucleic acid and lipid damage by oxidation [7], contributing to vascular and endothelial damage [5]. In diabetes, several interplaying factors lead to an increase in oxidative stress, including the accumulation of glycolysis intermediates, generation of advanced glycation end products (AGEs), activation of protein kinase C (PKC), activation of the polyol and hexosamine pathways, and mitochondrial dysfunction [8,9].

AGEs, which can be generated when excess glucose interacts with the amino acid groups of plasma proteins, can also lead to structural and functional alterations in endothelial cells and blood vessels, and the activation of pro-inflammatory pathways [8]. Endothelial cells are particularly susceptible to hyperglycemia-induced damage. AGEs and excess ROS can also lead to a decrease in nitric oxide (NO) and a reduction in the activity of the endothelial NO synthase and consequent loss of the NO-dependent vasodilatory response, which is a hallmark of diabetic endothelial dysfunction [9].

Regarding inflammation, cytokines, such as tumor necrosis factor α (TNF-α) and interleukin 1β (IL-1β), lead to the expression of cell adhesion molecules on endothelial cells. This induces an increase in circulating polymorphonuclear leukocytes and the release of matrix metalloproteinases, which cause the disruption of the blood–brain barrier and edema [6].

A decrease in mitochondrial enzymatic activity decreases antioxidant defenses, thus increasing oxidative stress and potentiating the effect of ischemia [10].

## 3. Current Prevention Strategies

Current stroke prevention strategies in patients with T2DM are based on controlling each of the risk factors. Therefore, lifestyle measures are of primordial importance. Intensive blood pressure control has also been shown to reduce the risk of cerebrovascular damage in people with T2DM [11]. The presence of T2DM alone places patients in higher cardiovascular risk categories, leading to the recommendation for stricter control of the lipid profile [12,13].

Additionally, it is recommended to implement antithrombotic strategies when indicated or anticoagulation for those with a cardioembolic source [14,15]. For those with significant carotid stenosis, revascularization should be considered [16].

The hallmark study UK Prospective Diabetes Study (UKPDS) in patients with recent onset of T2DM demonstrated that improved glycemic control was associated with very early benefits in reducing microvascular complications. Conversely, no immediate effect on macrovascular complications was seen. In the 10-year analysis, benefits in reducing macrovascular complications started to emerge, but only in the metformin arm. Patients on insulin and sulphonylureas only had clear benefits at 20 years [17,18]. Trials such as Action to Control Cardiovascular Risk in Diabetes (ACCORD), Action in Diabetes and Vascular Disease: Preterax and Diamicron Modified Release Controlled Evaluation (ADVANCE), and Veterans Affairs Diabetes Trial (VADT) sought to analyze the impact of aggressive glycemic control on macrovascular events in people with several years of DM and established or high risk for cardiovascular disease (CVD). The results obtained did not favor the use of strict glycemic targets in this group of patients to optimize cardiovascular outcomes [19]. An important consideration is that the more intensive glycemic control in these studies was attained at the cost of a twofold increase in the risk of severe hypoglycemia, which is a potent marker of high absolute risk of cardiovascular events and mortality [20]. A metanalysis of individual participant data from these four studies demonstrated a significant reduction in myocardial infarctions and major CVD events as a whole, but no difference in stroke, heart failure, or mortality between intensive and less intensive glycemic control [21]. These landmark studies need to be considered with the important caveat that drugs such as sodium–glucose cotransporter type 2 inhibitors (SGLT2is) and glucagon-like peptide 1 receptor agonists (GLP1-Ra) were not in clinical use at the time.

One might pose the following question: could stroke prevention depend not only on the degree of glycemic control, but also on the choice of antihyperglycemic drugs? This issue will be explored in subsequent sections of this text.

## 4. New Antihyperglycemic Drugs and Influence on Stroke

The treatment algorithm recommended for T2DM has undergone important changes in recent years. Until 2017, the American Diabetes Association (ADA) recommended the use of metformin as the first-line and a selection of the following drugs based on factors such as effectiveness, cost, and adverse effects [22]. In 2018, the ADA and the European Association for the Study of Diabetes (EASD) published a consensus report in which it was recommended, after metformin, preference for SGLT2is and GLP1-Ra in patients with established cardiovascular disease or chronic kidney disease [23]. In recent years, data have emerged to support the benefit of these two groups of drugs, even in patients not treated with metformin [24]. In this context, in 2022, the ADA and the EASD published a new consensus in which they recommend that drugs such as SGLT2is and GLP1-Ra can be used as first-line therapies in patients with established or high risk for CVD, independently of the background use of metformin [25].

The changes observed in the diabetes treatment algorithm largely result from the various cardiovascular outcome trials that have been mandatory according to the Food and Drug Administration since 2008. The initial aim of these trials was to demonstrate the cardiovascular safety of drugs for the treatment of DM before their introduction to the market. However, data have emerged demonstrating the cardiovascular and renal benefits of drugs such as GLP1-Ra and SGLT2is, justifying their preference [26]. In these trials, the following are frequently assessed: the risk of the three-point composite outcome of major cardiovascular events (3P-MACE)—non-fatal myocardial infarction, non-fatal stroke, and cardiovascular death, the risk of hospitalization for heart failure (HF), and risk of adverse renal events. Therefore, stroke is evaluated as a primary outcome only in conjunction with other events, not allowing the benefits of this outcome to be so directly inferred [27].

### 4.1. Glucagon-like Peptide 1 Receptor Agonists (GLP1-Ra)

Glucagon-like peptide-1 (GLP-1) is a peptide predominantly produced in enteroendocrine L cells in the distal ileum and colon. Its plasma levels increase within minutes after food ingestion. The GLP-1 receptor (GLP1-R) is expressed in the α and β cells of pancreatic islets, in the central and peripheral nervous system, heart, kidney, lung, and gastrointestinal tract. The activation of this receptor increases insulin release in a glucose-dependent manner, promotes resistance to apoptosis, increases β-cell survival, inhibits glucagon secretion, delays gastric emptying, diminishes food intake, and promotes greater glucose elimination through neural mechanisms [28].

Most of these drugs have demonstrated cardiovascular benefits, and are currently recommended as one of the preferred groups in people with T2DM and established or high risk of CVD and/or chronic kidney disease (CKD) [25].

As previously mentioned, in cardiovascular outcome trials (CVOTs), the composite of major cardiovascular events (3P-MACE) is frequently used. Several clinical trials to date have demonstrated a protective effect of GLP1-Ra regarding major cardiovascular events, namely LEADER (Liraglutide Effect and Action in Diabetes: Evaluation of Cardiovascular Outcome Results), SUSTAIN-6 (Semaglutide Unabated Sustainability in Treatment of Type 2 Diabetes 6), Harmony Outcomes with Albiglutide, REWIND (Researching Cardiovascular Events With a Weekly Incretin in Diabetes) with dulaglutide, and the AMPLITUDE-O trial (Effect of Efpeglenatide on Cardiovascular Outcomes) [29,30,31,32,33]. CVOTs with Lixisenatide, Exenatide, and oral semaglutide have so far demonstrated non-inferiority [34,35,36]. In these trials, the effect on the risk of stroke was also specifically reported as a secondary outcome, with the SUSTAIN 6 and REWIND studies showing a statistically significant reduction in the risk of fatal and non-fatal stroke (hazard ratio 0.61, CI 0.38–0.99 and 0.76 CI 0.62–0.94, respectively) [30,32]. In most of the remaining trials, there was also a trend toward a reduction in the risk of stroke, although without reaching statistical significance [3].

Several metanalyses were performed that provided data on GLP1-Ra’s influence on the risk of stroke. In a network metanalysis from 2019, GLP1-Ra (vs. placebo) was shown to significantly reduce non-fatal stroke (OR = 0.88, 95% CI (0.77, 0.99), *p* = 0.002) [37]. In 2020, Malhotra et al. found that GLP-1Ra could reduce nonfatal strokes (OR 0.84; 95% CI (0.76–0.94)) and all strokes (OR 0.84; 95% CI (0.75–0.93), *p* = 0.001) [38]. Subsequent metanalyses performed by Grewal et al. and Palmer et al. also corroborated a beneficial effect of GLP1-Ra on stroke prevention [39,40]. Wei et al. reported that a 17% reduction in the risk of stroke with GLP1-Ra (vs. placebo) (RR = 0.83, 95% CI (0.73, 0.95), *p* = 0.005) was at the cost of ischemic stroke (RR = 0.83, 95% CI (0.73, 0.95), *p* = 0.008) reduction, with no effect on hemorrhagic stroke (RR = 0.83, 95% CI (0.57, 1.20), *p* = 0.31) [41].

Most data suggest the prevention of recurrent stroke, but there are also data on primary prevention. A retrospective cohort study based on an insurance database in Thailand (1998 to 2018) sought to evaluate the effect of GLP1-Ra on the prevention of ischemic stroke in the Asian population with T2DM without established CVD. The authors conclude that prolonged use and higher doses of GLP-1-Ra were associated with a reduced risk of hospitalization for ischemic stroke among Asian patients with T2DM who did not have established atherosclerotic CVD [42].

A post hoc analysis of the SUSTAIN 6 and the PIONEER trials also supported semaglutide’s ability to reduce the risk of first stroke. This effect was mainly attributed to the reduction in small vessel occlusion [43].

Several mechanisms have been proposed for the neuroprotection conferred by GLP1-Ra, namely a neuroprotective effect with increases in neurogenesis and angiogenesis and decreases in apoptosis, oxidative stress, neuroinflammation, excitotoxicity, and the area of infarction [44]. Beneficial effects on atherosclerotic plaque have been supported by several preclinical studies [45,46], as well as by data suggesting that liraglutide decreases the medial thickness of the carotid intimal layer [47]. There also seems to be a role in the reduction in reactive oxygen species production and oxidative stress [41]. Preclinical studies suggest that liraglutide decreases inflammation in endothelial cells through calcium and AMP-activated protein kinase mechanisms and increases the expression of antioxidant genes downstream of protein kinase A and cAMP response element-binding protein [41]. GLP1-Ra was also shown to lead to a decrease in the inflammatory macrophage activation molecule sCD163, a decrease in the levels of TNF-α and IL-6 (pro-inflammatory), and an increase in adiponectin (anti-inflammatory) [48]. There may be an additional contribution to increasing neuronal energy supply through the positive regulation of GLUT4 involved in the supply of metabolic energy to fire neurons [41]. Recent metanalysis showed that the overall reduction in cardiovascular events with GLP1-Ra seems to be mediated in part by decreases in hemoglobin A1C [49,50]. In addition to all these effects, there are indirect benefits through action on various cardiovascular risk parameters, namely the modest improvement in lipid profile and blood pressure and a significant effect on weight [3].

When considering the introduction of GLP1-Ra, it is essential to take into account potential side effects that may limit their use. The side effects of GLP1Ra encompass gastrointestinal disturbances, pancreatitis, gallbladder disease, increased heart rate, and potential increased risk of medullary thyroid cancer (only documented in rodents) [51,52]. Recently, some concerns have been raised regarding its use in patients with HF with reduced ejection fraction (HF-rEF), which may be related to its effect on heart rate [53].

### 4.2. Sodium–Glucose Cotransporter Type 2 Inhibitors (SGLT2is)

Glucose only begins to be excreted in the urine when a glycemia threshold of around 180 mg/dL is exceeded, with a linear increase in glycosuria beyond this concentration. This threshold for glycosuria increases in patients with T2DM. SGLTs are responsible for transporting glucose across epithelial cells by active transport. The SGLT2 isoform is mainly found in the proximal convoluted tubule, being responsible for the reabsorption of over 90% of filtered glucose. Additionally, it is also found in much lower concentrations in the brain, liver, thyroid, and skeletal muscle. The pharmacological blockade of these transporters leads to an increase in glycosuria, allowing the excretion of 50–60% of filtered glucose [54,55].

Studies on cardiovascular outcomes have demonstrated the effectiveness of SGLT2is in reducing the incidence of hospitalizations for HF and the 3P-MACE composite, and most evidence suggests that the benefit on 3P-MACE may be limited to people with established CVD [56]. In general, observational studies have demonstrated the benefit of SGLT2is in both the primary and secondary prevention of 3P-MACE events, whereas RCTs have not, which is likely attributed to the different conditions of these types of studies [57].

Several mechanisms have been proposed for the cardiorenal benefits of SGLT2is, namely decreased blood pressure; modest weight loss; improvement of cardiac energy metabolism; prevention of inflammation, ischemia–reperfusion injury, and adverse cardiac remodeling; and inhibition of the sympathetic nervous system [58]. The decrease in glucose and sodium reabsorption achieved by the inhibition of SGLT2 leads to a decrease in HbA1c and global glucotoxicity. There is also a local effect of reducing glucose overload in tubular cells, which enhances the production of erythropoietin with consequent better oxygenation. With the increase in sodium excretion, there is an improvement in the reduction of intraglomerular pressure, a reduction in albuminuria, preservation of glomerular filtration rate, and a decrease in blood pressure. The increased excretion of glucose enhances the negative caloric balance that leads to weight loss and the excretion of uric acid [55].

In the main CVOTs with SGLT2i, the impact on stroke risk has been reported [59]. In early CVOTs, such as Empagliflozin Cardiovascular Outcome Event Trial in Type 2 Diabetes Mellitus Patients (EMPA-REG) [60], Canagliflozin Cardiovascular Assessment Study (CANVAS) [61], Dapagliflozin Effect on CardiovascuLAR Events (DECLARE-TIMI) [62], and Ertugliflozin Efficacy and Safety Cardiovascular Outcomes Trial (VERTIS CV) [63], there was a neutral effect on the risk of total stroke and no fatal stroke. In EMPA-REG, there was actually a tendency toward a higher risk of stroke without statistical significance (HR 1.18, CI 0.89–1.56). In this regard, it should be noted that, more recently, the observational cohort study in 11 countries, EMPagliflozin comparative effectiveness and Safety (EMPRISE), was published, which even suggests that empagliflozin may be associated with an 18% reduction in the risk of stroke compared with dipeptidyl peptidase 4 inhibitors (DPP4is) [64]. More recent data derived from the EMPRISE study have shown a similar risk of the composite outcome myocardial infarction or stroke when compared with GLP1-RA. The risk of stroke was evaluated as a secondary outcome and there was no statistically significant difference (HR 1.08, IC 0.95–1.22 for SGLT2i) [65]. Thus, although it has been proposed that there may be a contribution of the effect of hypotension and hemoconcentration justifying an increased risk of stroke with SGLT2i [59], it is more likely that it is an effect of chance.

Some authors argue that these CVOTs may have insufficient statistical power to test the effect of SGLT2i on reducing stroke risk, given that they mainly included patients with DM with established CVD other than stroke. Furthermore, nonfatal stroke is typically considered a component of the 3P-MACE composite outcome, rather than the primary outcome [59]. Other authors, however, suggest that SGLT2i reduces the 3P-MACE in a way unrelated to antiatherogenic effects, as there seem to be no discernible effects on MI or stroke incidence [66].

In a metanalysis published in April 2023, 36 clinical trials that reported on the effect of SGLT2is on the occurrence of stroke were taken into account, and no statistically significant difference was found between patients under SGLT2i vs. the control group, (RR: 0.97; 95% CI, 0.89–1.07, *p* = 0.56), even though SGLT2i use was associated with a statistically significant reduction in the risk of AF (RR: 0.87; 95% CI, 0.76–0.99, *p* = 0.03) [67]. A previous metanalysis also indicated neutrality of SGLT2i regarding stroke [37,40].

Lui et al. evaluated a population-based retrospective cohort of people with T2DM identified from Hong Kong electronic health records between 2008 and 2020. Patients who received SGLT2i or GLP-1RA were matched by propensity score matching. The authors did not find a significant difference between the SGLT2i and GLP1-Ra groups. They found, however, that patients with a history of stroke seemed to benefit more from the use of GLP1-Ra. There was a significantly higher risk of ischemic stroke among people on SGLT2is compared with GLP1-Ra users (HR 1.53, 95% CI 1.01–2.33, *p* = 0.044; IRR 1.52, 95% CI 1.02–2.27, *p* = 0.041). The risks of hemorrhagic stroke and cardioembolic stroke were similar for the groups treated with both pharmacological groups [68].

Regarding hemorrhagic stroke, there are data from two meta-analyses attributing a 50% reduction in the risk of hemorrhagic stroke to patients on SGLT2i vs. placebo [69,70]. There are several theoretical explanations for a possible benefit of SGLT2is on the risk of hemorrhagic stroke: a beneficial effect of blood pressure reduction, [68] increased low-density lipoprotein (LDL) cholesterol and triglycerides, reduced overload of small vessels and microhemorrhages, and “cerebrorenal” interaction [59]. A 4–10 mmHg reduction in systolic blood pressure has indeed been observed among both hypertensive and normotensive patients with T2DM using SGLT2is [71]. Some studies have suggested a relationship between a reduction in LDL and a higher risk of hemorrhagic stroke, but this is highly controversial and not supported by more recent analyses [72]. A beneficial effect of SGLT2is on kidney outcomes has been demonstrated consistently in several clinical trials and real-world studies [73]. There are several traditional risk factors shared between CKD and cerebrovascular disease. Uremia and changes in phosphocalcium metabolism can contribute to vascular medial calcification and endothelial damage, and CKD can precipitate certain stroke risk factors, such as AF [59].

A recent comprehensive metanalysis analyzed the differential effect of SGLT2is and GLP-1Ra on the risk of stroke. The authors report that, even though data from RCTs and CVOTs mostly favor GLP1-Ra, data from observational studies do not support a difference in the risk of stroke between the two pharmacological classes. Differences in study populations and lack of head-to-head comparisons in RCTs were suggested as potential explanatory factors. As such, the authors conclude that it is not linear whether, in the real-life setting, GLP-1Ra should be prioritized over SGLT2is for stroke prevention [74].

It is also worth considering that SGLT2is may be beneficial in stroke prevention in specific populations, such as those with HF with reduced ejection fraction (HF-rEF), AF, and more severe CKD. Among patients with HF-rEF but not with preserved ejection fraction (HFpEF), the initiation of SGLT2i versus DPP4i was associated with a lower risk of myocardial infarction or stroke (HR: 0.86 [0.75. 0.99]). In comparison with GLP1-Ra, this superiority of SGLT2is was not demonstrated [56]. In a nationwide database from Taiwan, medication with SGLT2is in T2DM patients with AF and without previous cardiovascular events was associated with a lower incidence of stroke and dementia [75]. A recent metanalysis of 10 RCTs suggested that the effects of SGLT2is on stroke, HF, and cardiovascular death prevention was greater for patients with more severe CKD. Stroke risk reduction was only found in patients with a lower glomerular filtration rate (GFR), while in patients with a GFR >80 mL/min/1.73 m^2^, a higher risk was found in patients under SGLT2i vs. placebo [76].

It is also important to mention a metanalysis by Sayour et al., which sought to evaluate whether there was an influence of SGLT2 receptor selectivity on cardiovascular outcomes. The authors concluded that, in high-CV-risk T2DM patients, dual SGLT2/1 inhibition reduced stroke risk, but had limited additional benefits [77].

To conclude this section, it is paramount to be aware of the potential disadvantages of this drug class when contemplating their introduction to patients with T2DM. Side effects of SGLT2i include elevated ketone body levels and euglycemic ketoacidosis (rare in T2DM), genital mycotic infections, volume depletion, acute kidney injury, and possibly, although controversial, Fournier gangrene. Canagliflozin may also increase the risk of lower-extremity amputation and bone fractures, although such effects have not been reported with other SGLT2is [52,78,79].

### 4.3. Dipeptidyl Peptidase 4 Inhibitors (DPP4is)

Dipeptidyl peptidase 4 is an enzyme that breaks down incretin hormones, such as GLP1 and glucose-dependent insulinotropic polypeptide (GIP). DPP4is work by blocking this enzyme, thus preventing the breakdown of incretin hormones. GLP1 is believed to be the major player in mediating the therapeutic effect of DPP4is [80].

These drugs have also been subjected to cardiovascular outcome trials, but generally demonstrating neutral results [81,82].

Regarding stroke specifically, the metanalyses carried out to date have, as expected, corroborated the neutrality of these drugs in the risk of stroke, highlighting the beneficial effect of GLP1-Ra [83,84]. Although DPP4is have a similar mechanism of effect to GLP1-Ra, these drugs do not cross the blood–brain barrier, do not induce an increase in plasma GLP1, and have a weak effect on reducing infarct volume [83].

#### 4.3.1. Tirzepatide

Tirzepatide is a dual GLP-1 and GIP receptor agonist, approved by the Food and Drug Administration (FDA) and later by the European Medicines Agency (EMA) for T2DM [58,85].

The tirzepatide versus insulin glargine in type 2 diabetes and increased cardiovascular risk: a randomized, open-label, parallel-group, multicenter, phase 3 trial (SURPASS-4) has been completed, demonstrating a great reduction in HbA1c, less risk of hypoglycemia, and cardiovascular safety with tirzepatide vs. glargine [86]. SURPASS-CVOT is still ongoing and will be able to better clarify the advantages of this drug and its impact on CVD [87].

#### 4.3.2. Finerenone

Finerenone is a non-steroidal mineralocorticoid receptor antagonist that has demonstrated cardiovascular benefit in patients with T2DM and risk of CVD. It is currently recommended by the ADA in people with CKD to reduce the risk of CVD and the progression of kidney disease in those with albuminuria [88].

Although the drug has effectively shown a benefit in the 3P-MACE composite outcome in the Finerenone in Chronic Kidney Disease and Type 2 Diabetes: Combined FIDELIO-DKD and FIGARO-DKD Trial Programme Analysis (FIDELITY) pooled analysis, it has not yet been shown to specifically prevent stroke [89,90].

Table 1 provides a summary of the evidence regarding the more recently introduced therapies, that underwent CVOTs, on 3P-MACE in general and on stroke in particular. 

## 5. What about “Older” Pharmacological Options?

CVOTs only became mandatory from 2008 onwards, so for drugs approved before this year, there are not as much solid data regarding cardiovascular outcomes. Still, other types of analyses are available that provide some information.

Metformin is an older drug of established relevance in the treatment of T2DM. There are data suggesting cardiovascular benefits of this drug. In the UKPDS, the metformin arm had a 30% lower risk of cardiovascular complications compared with the diet group, [18] which was also seen in the 10-year follow-up after the trial [91]. A 2017 metanalysis documented the benefit of metformin on all cardiovascular endpoints except stroke, but without reaching statistical significance [92]. In 2019, Han et al. and later Zhang et al. published metanalyses that demonstrated reduced cardiovascular risk with metformin [93,94]. Regarding stroke, there are data suggesting a beneficial effect of metformin, particularly if there is a sustained use pre-event that is maintained afterward [95]. Taking metformin prior to stroke seems to be associated with lower stroke severity and better functional outcomes in patients with small-vessel disease [96].

Within the group of drugs approved prior to the need for CVOTs, glitazones have received the most attention in the context of stroke and are recommended in current guidelines [25,52]. In experimental models, treatment with peroxisome proliferator-activated receptor gamma (PPARγ) agonists improved insulin sensitivity and inhibited the development of atherosclerosis [27]. Additionally, treatment with pioglitazone reduced the area of post-stroke hemorrhagic transformation [97]. In clinical studies, most evidence is of the prevention of recurrent stroke. In the PROactive study, pioglitazone reduced the risk of recurrent stroke by 24% (HR = 0.53, 95% CI = 0.34–0.85; *p* = 0.0085), but not of the first stroke [98]. In the Insulin Resistance Intervention after Stroke (IRIS) trial, pioglitazone was effective in the secondary prevention of ischemic stroke in patients without DM with insulin resistance [99]. In a Korean publication, a benefit was reported in preventing the first stroke event (odds ratio 0.69, 95% CI (0.60–0.80)), with the greatest benefit in patients with hypertension, obesity, smoking, and a sedentary lifestyle [100].

Sulphonylureas stimulate insulin secretion by closing potassium channels in pancreatic beta-cells independently of plasma glucose levels. The cardiovascular safety of sulfonylureas is controversial. Two metanalyses showed a significantly higher risk of stroke for SUs than for comparator drugs in patients with T2DM [101,102]. In a subsequent population-based cohort study with almost 100,000 patients with T2DM, sulphonylureas use was associated with higher risks of stroke, cardiovascular mortality, and all-cause mortality compared with metformin [103]. A more recent retrospective study with a comparable size demonstrated similar findings [104]. These results suggest that sulfonylureas should be considered carefully or even avoided in patients with high cardiovascular risk.

## 6. Clinical Implications of Current Data

As previously mentioned, since 2022, the ADA and EASD consensus, and also the current ADA Standards of Care, propose that GLP1-Ra and/or SGLT2i be included in people with T2DM and established or high risk for CVD, HF, and/or CKD to reduce the cardiovascular risk [25,52]. However, given that there is evidence suggesting a greater benefit of GLP1-Ra in atherosclerotic disease and stroke, currently, several authors propose the preferential use of this pharmacological group in patients with a previous history of stroke [49,58]. Pioglitazone is also a drug to be considered by the ADA and the EASD after the previously mentioned drugs in patients with established atherosclerotic CVD [25,52]. Given the relatively frequent coexistence of HF in these patients, it is necessary to take into account HF when considering initiating glitazones and, more recently, the doubt that has also been raised regarding the safety of GLP1-Ra in patients with HF-rEF [53]. Considering all the above-mentioned data, in patients with a history of atherosclerotic disease and/or ischemic stroke, especially without HF, a logical order of introduction of drugs could be GLP1-Ra, followed by SGLT2i, and later pioglitazone. However, in patients with stroke and a history of HF-rEF, advanced CKD, and/or hemorrhagic stroke, the evidence for selection is not as strong and a SGLT2i will often be placed as a first option.

As for the primary prevention of stroke, besides the importance of adequate glycemic control, the specific targeting of obesity, CKD, and other CV risk factors may also influence antidiabetic drug choices. The discussion of the influence of different pharmacological classes on CVD and CKD has been explored in previous sections of this text. As for targeting obesity, both GLP1-Ra and SGLT2i have a beneficial effect on weight, but the GLP1-Ra may be associated with a greater reduction, particularly semaglutide. Tirzepatide is also associated with an important weight reduction [52].

**Figure 1:**
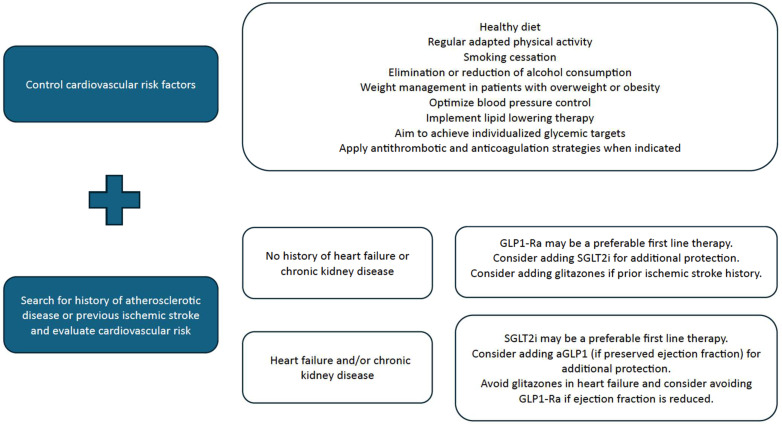
Summary of proposed recommendations for stroke prevention in type 2 diabetic patients. GLP1-Ra—Glucagon-like peptide 1 receptor agonists; SGLT2i—Sodium–glucose cotransporter type 2 inhibitors.

Figure 1 provides a summary of the proposed recommendations for stroke prevention in type 2 diabetic patients based on current data.

## 7. Conclusions

In recent decades, CVOTs have brought important data on the role of drugs designed to treat DM in the prevention of cardiovascular events. There is still much to learn, however, on the specific role of these drugs in stroke prevention. Nevertheless, data suggest that GLP1-Ra has a particularly beneficial effect on ischemic stroke. As for hemorrhagic stroke, the available information is less clear, but SGLT2is may be particularly beneficial.

## 8. Future Perspectives

In recent years, the algorithm of treatment of T2DM has suffered tremendous alterations and currently is not exclusively focused on glycemic control, but also on other goals, such as cardiorenal risk and weight management. Nevertheless, there is still much that can be perfected regarding individual goal definition and treatment personalization.

In the near future, studies on newer pharmacological classes are also expected to emerge and to add useful information. The above-mentioned SURPASS-CVOT is a double-blind, active-controlled CV outcomes trial which is evaluating the cardiovascular safety and efficacy of tirzepatide vs. dulaglutide. As in previous trials, the MACE composite is the primary outcome and data on stroke may be available as a secondary outcome [81]. Most patients with diabetes will be at risk for different types of cardiovascular events (myocardial infarction, ischemic stroke, hemorrhagic stroke, peripheral artery disease, …) and will also have several risk factors that may limit the utilization of specific drug classes. As such, it is paramount to stimulate stakeholders to evaluate the effect on each of the macrovascular complications in a detailed manner, for instance, having specific trials with stroke as a primary outcome. In the era of personalized medicine, the exponential increase in knowledge regarding new therapies will hopefully allow clinicians to further take into account the individual characteristics of each patient in the selection of the most adequate treatment combination.

## Figures and Tables

**Table 1:** Summary of data on the effect on major adverse cardiovascular events and stroke from novel therapies for the treatment of type 2 diabetes mellitus.

	Effect on 3P-MACE	Effect on Stroke
Glucagon-like Peptide 1 Receptor Agonists (GLP1-Ra)	RCTs show a benefit of subcutaneous liraglutide, dulaglutide, semaglutide, and efpeglenatide [29,30,31,32,33] and non-inferiority for lixisenatide, exenatide, and oral semaglutide [34,35,36].	Some evidence of a benefit in RCTs where it was evaluated as a secondary outcome [30,32]. Several metanalyses support a stroke risk reduction [37,39,40], particularly on isquemic stroke. No effect on hemorrhagic stroke was seen [41].
Sodium–glucose cotransporter type 2 inhibitors (SGLT2i)	Demonstrated effectiveness in reducing the incidence of hospitalizations for HF and the 3P-MACE composite in CVOTs, although mostly limited to patients with established CVD [56]. Observational studies have demonstrated the benefit of SGLT2i in both primary and secondary prevention of 3P-MACE events [57].	In CVOTs, no statistically significant difference was observed regarding stroke [60,61,62,63]. Metanalyses also suggest neutrality [37,40,67]. Data from observational studies do not support a difference in the risk of stroke compared with GLP1-Ra [74]. May be beneficial in stroke prevention in specific populations, such as those with HF with reduced ejection fraction [56], atrial fibrillation [75], and more severe chronic kidney disease [76].
Dipeptidyl Peptidase 4 Inhibitors (DPP4is)	Neutral results in CVOTs [81,82].	Neutral results in CVOTs [83,84].
Tirzepatide	Non-inferiority vs. glargine [86]. CVOT is ongoing [87].	CVOT is ongoing [87] and may provide data.
Finerenone	Benefit in 3P-MACE [89,90].	Not shown to specifically prevent stroke in CVOTs [89,90].

CVD—cardiovascular disease; CVOTs—cardiovascular outcome trials; DPP4is—Dipeptidyl peptidase 4 inhibitors; GLP1-Ra—Glucagon-like peptide 1 receptor agonists; HF—heart failure; MACE—major adverse cardiovascular events; RCTs—randomized controlled trials; SGLT2i—Sodium–glucose cotransporter type 2 inhibitors.
